# Fearlessness as an Underlying Mechanism Leading to Conduct Problems: Testing the INTERFEAR Model in a Community Sample in Spain

**DOI:** 10.3390/children11050546

**Published:** 2024-05-03

**Authors:** Kostas A. Fanti, Ioannis Mavrommatis, Beatriz Díaz-Vázquez, Laura López-Romero, Estrella Romero, María Álvarez-Voces, Olivier F. Colins, Henrik Andershed, Nicholas Thomson

**Affiliations:** 1 Department of Psychology, Faculty od Social Sciences and Education, University of Cyprus, P.O. Box 20537, Nicosia 1678, Cyprus; mavrommatis.ioannis@ucy.ac.cy; 2 Instituto de Psicoloxía (IPsiUS), Universidade de Santiago de Compostela, 15782 Santiago de Compostela, Spain; beatrizdiaz.vazquez@usc.es (B.D.-V.); laura.lopez.romero@usc.es (L.L.-R.); estrella.romero@usc.es (E.R.); mariaalvarez.voces@usc.es (M.Á.-V.); 3 Department of Special Needs Education, Faculty of Psychology and Educational Siences, Ghent University, Dunantlaan 1, 9000 Gent, Belgium; olivier.colins@ugent.be; 4 School of Behavioural, Social and Legal Sciences, Örebro University, 701 82 Örebro, Sweden; henrik.andershed@oru.se; 5 Department of Surgery and Psychology, Virginia Commonwealth University, Richmond, VA 23284, USA; nicholas.thomson@vcuhealth.org

**Keywords:** fearlessness, conduct problems, parenting, callous–unemotional traits

## Abstract

Conduct problems (CP) in childhood and adolescence have a significant impact on the individual, family, and community. To improve treatment for CP, there is a need to improve the understanding of the developmental pathways leading to CP in boys and girls. Prior research has linked the child’s fearlessness and callous–unemotional (CU) traits, as well as experiences of parental warmth and punitive parenting, to CP. However, few studies have tested the interplay of these factors in contributing to future CP development. The present study aimed to test the InterFear model, which suggests that fearlessness in early childhood leads to CP through an indirect pathway involving low positive parenting, high negative/punitive parenting, and callous–unemotional (CU) traits. The sample included 2467 Spanish children (48.1% girls; *M*age = 4.25; *SD* = 0.91), followed up across a five-year period. Besides a direct association between fearlessness in early childhood and future CP, the results found an indirect pathway whereby fearlessness reduces positive parenting and increases punitive parenting, which contributes to the development of CU traits and sets the stage for CP in later childhood. The specific indirect effect from fearlessness to CP via CU traits accounted for most of the variance, suggesting the existence of a temperamental pathway independent of parental variables. Further, two additional indirect pathways, exclusive of fearlessness, were identified, which started with low parental warmth and positive parenting, leading to CP via CU traits. These findings support the InterFear model, demonstrating multiple pathways to CP with the involvement of fearlessness, parenting practices, and CU traits. This model might play a pivotal role in the development of targeted prevention and intervention strategies for CP.

## 1. Introduction

Conduct problems (CP) are one of the most frequent causes for which psychoeducational and clinical assistance is needed during childhood and adolescence [1,2,3]. CP denotes a range of manifestations that include aggression, oppositionality, vindictiveness, anger, or violations of age-appropriate norms [4], which are predictors of enduring antisocial behavior and criminality over the course of a lifetime [5]. Child variables, such as fearless temperament or psychopathic traits (e.g., lack of empathy, callous–unemotional [CU] traits), and family variables (e.g., parental warmth or punitive parenting), have been extensively related to CP in numerous longitudinal studies. However, there has been limited research on the interrelationships of these factors and their role in the development of CP. In this regard, the study by Fanti et al. [6], which is based on a sample from the longitudinal study SOFIA (Social and Physical Development. Interventions and Adaptation) collected in Sweden, represents a pioneering line of research that proposes a developmental model (i.e., InterFear model) in which fearlessness is connected to CP through different family (i.e., harsh parenting, low warmth, parent–child conflict) and individual variables (i.e., CU traits and anxiety). The current study’s objective is to test the InterFear model in a different country, using an ongoing longitudinal study in Spain, to provide cross-national evidence that can enhance the understanding of the development of CP.

### 1.1. Back to the Origin: The Role of Fearlessness

Fearlessness is intricately connected to fearful arousal, signifying a predisposition to display reactive fear and heightened sensitivity to threats when faced with potential danger, harm, or uncertainty in the environment (i.e., trembling, freezing, and facial expressions of fear [7]). A fearless temperament typically implies the tendency to react with lower arousal to unfamiliar circumstances, people, and negative emotional stimuli, including the negative consequences of one’s behavior (e.g., punishment [8]). This lower arousal also includes a reduction in the perception and sensitivity to fear signals from others, which may cause difficulties in learning empathy and guilt that, in turn, may cause socialization problems [9,10,11,12]. Thus, prosocial learning difficulties and insensitivity to punishment might lead children with a fearless temperament to seek out stimulating new situations, such as dangerous or antisocial behaviors that may increase the risk of developing CP [13,14,15,16,17].

**Figure 1:**
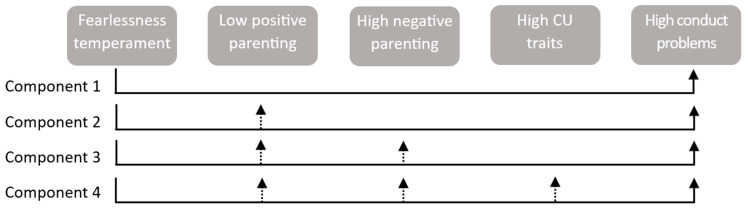
The InterFear model (adapted from Fanti et al. [6]). Note. The solid line in component 1 signifies the direct connection between fearless temperament and elevated CP. The other components of the model depict indirect effects, indicating that this relationship is influenced by intermediate family and individual variables, highlighting the complex interplay between temperament, family dynamics, and individual factors in the development of conduct problems.

In the InterFear model, childhood fearlessness stands as the foundational precursor to CP, forming the bedrock of this developmental framework. Fearlessness serves as the initial trigger (see Figure 1, component 1), setting the stage for CP through a series of interconnected components. Low positive parenting, parental negativity, and high CU traits (see components 2, 3, and 4 in Figure 1) act as mediators in the association between fearlessness and CP. Fearlessness diminishes positive parental interactions, thereby intensifying parental negativity. This heightened negativity, in turn, amplifies individual variables linked to CU traits, ultimately propelling the development of CP. Additionally, there is a direct pathway from fearlessness to CP (component 1), contributing significantly to the manifestation of behavioral problems.

### 1.2. Parenting Practices and CP

Parenting practices have been widely studied as predictors of children’s future positive or maladjusted behavior, mostly in interaction with the child’s unique characteristics. A warm parenting style is characterized by a strong presence of positive affect, dedication, and a sense of closeness to the child [18,19]; hence, it includes parental behaviors that are affectionate, comforting, caring, accepting, and supportive of the child [20]. Aligned with appropriate discipline (i.e., setting clear rules and limits), parental warmth constitutes the baseline of positive parenting, which has been shown as a protective factor against future CP [21,22,23,24,25], overall influencing the child’s positive adjustment [26,27,28]. 

Research has previously examined the mediating role of parental warmth between fearlessness and CP. Some longitudinal studies suggest that high fearlessness is negatively related to warm parenting and that the presence of warm parenting plays a mediating role between high levels of fearlessness and decreases in future CP [29]. Yet, due to low arousal levels, which are the core of fearless temperament, fearless children may be less sensitive to parental efforts and consequently might continue engaging in problematic behavior even in the face of punishment [30,31]. This, in turn, may lead to decreased parental warmth and evoke harsh and punitive parenting practices overall [6].

Punitive parenting, characterized by inflexible rules, verbal or physical hostility, and extensive punishment (i.e., harsh parenting), has been proposed as a risk factor for the development of externalizing problems during childhood in various longitudinal studies (e.g., [25,32,33]). Also, the effect of punitive parenting might depend on other variables, such as child temperament (e.g., fearlessness). Agreeing with this suggestion, prior work showed that children exhibiting a fearless temperament are more prone to demonstrate increases in CP behaviors with the experience of negative parenting [29,34]. When studying whether parental warmth can mitigate the adverse impacts of punishment on psychological and behavioral adjustment, mixed results have been reported. Whilst some studies found a moderating effect of warmth [35,36,37], others found no effect of warmth in the association between parental punishment and child externalizing problems [38,39,40]. The combination of both parental warmth and punitive practices has given rise to one of the important theories regarding the emergence of child CP: Patterson’s coercion model [41]. This model proposes an indirect pathway where ineffective parenting practices (i.e., harsh parenting and low warmth), mostly resulting from a child’s difficult temperamental style, can result in coercive interactions and conflict between parents and children. Exchanges based on a coercive relationship within the dyad can reinforce children’s disruptive behavior as they learn to engage in social interactions in a coercive way [41].

### 1.3. The Role of Children’s CU Traits

CU traits represent a developmental extension of the affective facet of psychopathy, characterized by shallow affect and lack of empathy [13,42]. Empathy deficits stand out as a clear indication of CU traits (e.g., lack of guilt, remorse) [42,43,44,45], which are intricately connected to violence, psychopathy, and arrest even after controlling for disruptive behaviors [46]. In addition, prospective studies indicate that children characterized by CU traits tend to have more severe and stable forms of CP [47,48]. 

At their core, etiological models of CU traits assert that this personality characteristic stem from diminished basal physiological functioning and arousal [49]. Accordingly, individuals with elevated CU traits may exhibit reduced efficiency in processes that typically inhibit behavior in response to punishment or threat cues or that would motivate adaptive, norm–compliant behavior [13,14,50,51]. Longitudinal evidence has begun to link child fearlessness with the emergence of CU traits, supporting these theoretical positions [52,53,54,55]. Part of this evidence is the core of a new theoretical framework, the Sensitivity to Threat and Affiliative Reward (STAR) model [31], which postulates that CU traits emerge from deficits in sensitivity to threat (i.e., fearlessness) and affiliative reward. Moreover, the findings support the relationship between fearlessness and CU traits even after controlling for other key variables like harsh parenting [29]. The contribution of parental practices to the development of CU traits has also been studied. For example, there is a line of research suggesting that CU traits develop independently of parental practices [56,57]. These results indicate that children with CU traits have a greater insensitivity to punishment, which reduces the effectiveness of parenting in preventing CP [6,58]. This leads to a failure of moral and socio-emotional development leading to lower prosocial behaviors and higher levels of CP [59,60], which may result in a more treatment-resistant group that needs tailored interventions [61]. 

Finally, another possible scenario is that punitive and harsh parenting causes children to have deficits in conscience development. This would be due to fewer learning opportunities for children with low warmth and harsh and punitive parents [62]. This hypothesis is derived from Patterson’s Coercive Model [41], which proposes that parenting practices characterized by harsh parenting and low warmth may contribute to increases in antisocial behavior through experiences of conflictual parent–child relationships. We propose that low insensitivity and limited response to punishment, associated with fearlessness, would lead to a paradigm shift in the parenting style, increasing the likelihood of coercive and harsh punishment styles in an effort to control their children’s misbehavior [63] at the expense of warmth approaches. This would deprive children of a warm relationship where mechanisms of socio-emotional learning and moral learning could be developed, eventually leading to the continuation of negative behaviors after punishment [31]. Nevertheless, some researchers (e.g., [24]) consider that the combined association between the presence of harsh punitive parenting and low parental warmth is not so important as the absence of warmth in parenting, which would particularly trigger CP in children with CU traits. Based on the foregoing, the model tested in the present study intends to align with previous research, indicating that the absence of positive parenting, along with the rise of punitive parenting, increases the risk for later CU traits [34,54,64,65], which increases subsequent CP. 

### 1.4. Current Study

This study aims to evaluate the applicability of the InterFear model proposed by Fanti et al. [6] within a community population of children in Spain. To accomplish this aim, we will investigate the direct and indirect longitudinal connections among individual factors, specifically fearlessness and CU traits, as well as parental practices (i.e., positive parenting, parental warmth, and punitive parenting), in relation to future CP. Overall, we anticipate similar results as those reported in the original study [6]: Fearlessness is expected to emerge as an early precursor of future CP (Figure 1, component 1). We anticipate that fearlessness would lead to diminished parental warmth and positive parenting, contributing to the development of CP (Figure 1, component 2). The decline in parental warmth and positive parenting due to fearlessness is anticipated to be linked to an escalation in punitive parenting, increasing the risk of future CP (Figure 1, component 3). Lastly, we expect to identify a pattern from fearlessness to low positive parenting/low warmth and to increased punitive parenting, contributing to the development of CU traits that ultimately promote CP (Figure 1, component 4). Since Fanti et al. [6] found that the most important indirect pathway to CP was through CU traits and not anxiety, we did not include anxiety in the model; hence, only CU traits were included as the final mediator in the model. Beyond the hypothesized associations, two possible pathways for the development of CP are also hypothesized according to the study by Fanti et al. [6]: (1) A temperamental pathway that begins with fearlessness and leads to future CU traits and CP independently of parental variables and (2) an environmental pathway starting with low warmth/low parental positivity that results in increased punitive parenting that leads to future CP independently of CU traits.

## 2. Method

### 2.1. Participants

Data for the present study were collected in the first five waves of the Estudio Longitudinal para una Infancia Saludable (Longitudinal Study for a Healthy Childhood; [ELISA]), an ongoing longitudinal study conducted in Galicia (NW Spain) aimed at examining the behavioral, emotional, and psychosocial functioning from early childhood onwards. Data collection started in 2017 (T1) with an initial sample of 2467 children (48.1% girls; 93.9% Spanish) aged 3 to 6 years (*M*age = 4.25; *SD* = 0.91). Children were enrolled in 57 public (79.2%), 13 charter (18.1%), and two private (2.8%) schools located in predominantly working-class communities. Information was provided through 2266 parents’ reports (87.2% mothers) and 2420 reports from preschool teachers. Based on parents’ academic level, 47.4% of mothers and 31.2% of fathers, respectively, completed higher education, around 29% of parents completed vocational training, and 23.7–39.8% completed compulsory education. At the time of the first data collection, 77.2% of the mothers and 92.4% of the fathers were actively working. 

Subsequent follow-up studies were conducted in 2018 (T2), 2019 (T3), 2021 (T4), and 2022 (T5), encompassing a 5-year period. At T2, the initial sample was increased by 361 participants (51.5% boys, aged 3 to 5; *M*age = 3.77; *SD* = 0.87) from a specific area within the same region not covered in T1. It resulted in a final sample of 2712 children (51.8% boys; *M*age = 5.12; *SD* = 1.07), of which 2354 were from T1 (95.4%) and 358 were from the new sample. Information was provided by 2346 parents and 2522 teachers. At T3, information was available for 2628 participants (51.6% boys; *M*age = 6.11; *SD* = 1.07), 2288 from T1 (92.7%), and 340 from the T2 new sample (94.2%). Data were provided by 2105 parents and 2346 teachers. At T4, data were collected in a sample of 1968 children (51.8% boys; *M*age = 8.21; *SD* = 1.07), of which 1693 were from T1 (68.6%) and 275 (76.2) from T2. Data were collected through 1291 parents’ reports and 1426 teachers’ reports. At T5, data were available for 2135 participants (51.1% boys; *M*age = 9.16; *SD* = 1.06), 1858 from T1 (75.3%) and 277 from T2 (76.7%). Information was provided by 1603 parents and 1675 teachers. 

### 2.2. Attrition Analyses

Comparisons were conducted between participating children with data provided by parents and/or teachers in all data collections (*n* = 1709; 60.3%), children who missed one follow-up (*n* = 509; 18%), children who missed two or three follow-ups (*n* = 545; 19.2%), and those with available data just in the initial data collection, whether it was T1 or T2 (*n* = 69; 2.4%). Results revealed no significant differences in terms of sex. There were significant differences based on age at T2, *F*(3, 2339) = 13.42, *p* < 0.001; T3, *F*(2, 2249) = 13.37, *p* < 0.001; T4, *F*(2, 1251) = 5.56, *p* < 0.01; and T5, *F*(2, 1614) = 19.82, *p* < 0.001. There were also significant differences in terms of SES, *F*(2, 2576) = 24.43, *p* < 0.001, with higher SES in families with data available in all data collections. Regarding the study variables, there were significant differences in fearlessness reported by parents, *F*(3, 2243) = 6.66, *p* < 0.001, and teachers, *F*(3, 2415) = 3.50, *p* < 0.05, with lower fearlessness in children with available data in all waves of the study. No significant differences were identified for parenting variables, CU traits, and CP. 

### 2.3. Measures

A multi-informant approach was followed, with parents and teachers reporting on the child’s fearlessness (T1) and CP (T5). Parenting variables (T2 and T3) and CU traits (T4) were reported by parents. Both parents and teachers were asked to provide information about the child at the current moment or in the past months. The total scores were rendered by computing the mean scores. All measures have been used in previous research, supporting their internal consistency and construct validity (e.g., [6,15,52,66,67].

***T1 Fearlessness:*** Fearlessness was measured with The Child Fearlessness Scale [68], reported by parents and teachers. This scale consists of six items (e.g., “He/she does not seem to be afraid of anything”, “He/she does not seem to get scared when someone is mad at him/her”) scored on a four-point response scale, ranging from 1 (does not apply at all) to 4 (applies very well). In the current study, the scale showed good to excellent levels of internal consistency for parents (*α* = 0.85) and teachers (*α* = 0.92).

***T2 Warmth and Positive Parenting:*** Parental warmth was measured with 6 items based on the Warmth subscale from the Child Rearing Scale [69]. The items (e.g., “You express affection by hugging, kissing, and holding your child”, “You have warm, close times together with your child”) were scored by parents on a 5-point scale ranging from 1 (never) to 5 (very often) and showed good levels of internal consistency (*α* = 0.81). Positive parenting was measured with the Alabama Parenting Questionnaire for Preschoolers (APQ-Pr; [70]). This scale is composed of 42 items adapted from the original APQ measure [71]. In the current study, the 12-item scale of positive parenting was used. The items (e.g., “Praise your child when he/she behaves well”, “Ask your child about his/her day in school”) were reported by parents on a 5-point scale ranging from 1 (never) to 5 (always), with good internal consistency (*α* = 0.74).

***T3 Punitive Parenting:*** Punitive parenting was assessed via 3 items (e.g., “Slap your child when he/she has done something wrong”, “Hit your child with a slipper or other object when he/she has done something wrong”) also from the APQ-Pr [70]. The items were rated by parents in the aforementioned 5-point scale, from 1 (never) to 5 (always) and showed acceptable levels of internal consistency (*α* = 0.64).

***T4 CU Traits:*** CU traits were assessed with the parent version of the Child Problematic Traits Inventory (CPTI; [68], a 28-item measure intended to measure Grandiose–Deceitful, CU, and Impulsive–Need of stimulation traits in 3- to 12-year-old children. For the purpose of the current study, only the CU dimension was used. CU traits consist of 10 items (e.g., “Never seems to have bad conscience for things that he or she has done”, “Seldom express sympathy for others”), scored by parents on a 4-point scale from 1 (does not apply at all) to 4 (applies very well), with good levels of internal consistency (*α* = 0.87).

***T5 CP:*** Parents and teachers independently rated 10 CP items (e.g., “Has been very angry” and “Has beaten, torn, shoved, kicked, or thrown something on others without a reason”), which were developed based on DSM criteria for oppositional defiant disorder and conduct disorder [68]. Items were scored using a 5-point response scale ranging from 1 (never) to 5 (very often). The internal consistency of this scale was good for parents (*α* = 0.87) and excellent for teachers (*α* = 0.92).

### 2.4. Procedure

This study was approved by the Bioethics Committee at the University of Santiago de Compostela, the former Spanish Ministry of Economy and Competitiveness, and the Ministry of Science and Innovation. Firstly, the heads of 126 public, charter, and private schools were contacted to explain study details and request collaboration. Once the school agreed to collaborate, families were contacted and invited to participate. Families who agreed (around 25.50% per school) filled out an active consent form, which entailed completing a questionnaire about their child once a year and the approval of teachers’ participation, also filling out a questionnaire on each data collection. The principal caregiver (>85% mothers) completed the questionnaire by paper or via a secured web platform (online version). One teacher completed questionnaires for all children in his or her classroom with parental authorization provided via the written consent form. Most of the teachers accessed and completed the questionnaire using the online version. In both paper and online versions, confidentiality was ensured with a personal keycode to access and identify the questionnaires. In all waves of the study, data were collected during the spring to ensure that teachers had spent at least six months with the child before rating the questionnaire items. Overall, participants had one month to complete the questionnaire. Reminders were sent, first through the school and then by e-mail. Neither teachers nor parents received any monetary compensation for their participation. Instead, as a reward for their collaboration, all the participating schools received in T1 a set of child educational games. A draw of several sets of books and educational games, valued between EU 50 and 100, was carried out at the end of T3 for both families and schools. At T4 and T5, parents received a report of results about their child’s competencies, with suggestions for improvement, based on their responses to the questionnaire. Finally, formative talks for teachers and families were delivered upon request in all waves of the study.

### 2.5. Analysis Plan

**Figure 2:**
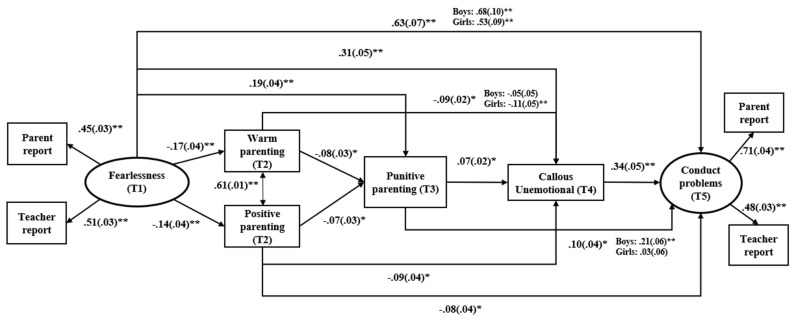
Structural Equation model testing the InterFear theoretical model. ** = *p* < 0.001; * = *p* < 0.05.

As demonstrated in Figure 2, the proposed theoretical model investigated longitudinal direct and indirect associations across five waves of measurement. Within the structural equation path model (SEM), the majority of measures were observed, whereas T1 fearlessness and T5 CP were latent variables, taking into account both teacher and parent reports. To examine the Interfear model, all possible indirect pathways were tested, starting from fearlessness to T2 warm and positive parenting, to T3 punitive parenting, to T4 CU traits, and eventually to T5 CP (see MacKinnon et al. [72] intervening effect method). To test for the fit of the SEM, we tested the Root Mean-square Error of Approximation (RMSEA < 0.06), Standardized Root Mean Residual (SRMR < 0.08), and the Comparative Fit Index (CFI > 0.95). Full Information Maximum Likelihood Estimator was used for the analysis to estimate missing data. To test for gender moderation, we used Little’s [73] statistical guidelines (i.e., a multi-group path model), which suggests a comparison of a constrained model (i.e., structural paths and correlations are constrained to be equal across genders) to an unconstrained model (i.e., associations were freely estimated across genders) using the chi-square difference test.

## 3. Results

### 3.1. Descriptive Statistics

Means, standard deviations, and bivariate correlations are shown in Table 1. As shown in Table 1, the associations between parent-reported fearlessness and warm/positive parenting were not significant. Importantly, time 1 (T1) fearlessness was correlated with all longitudinal assessments, with a higher association identified between fearlessness and T5 CP. Warm and positive parenting (T2) were negatively associated with CU traits and parent-reported CP. Punitive parenting (T3) was negatively associated with warm parenting and positively related to CU traits and CP. T4 CU traits were significantly associated with both parent and teacher-reported CP. 

### 3.2. Direct Effects

The SEM fitted the data well, *χ*^2^_(17, *N* = 2824)_ = 28.91, *p* < 0.001; *RMSEA* = 0.04 (RMSEA CI: 0.03|0.05), *SRMR* = 0.02, *CFI* = 0.96. The factor loadings for the Fearlessness, which were 0.45 and 0.51, and the CP, which were 0.71 and 0.48, latent factors were significant. Figure 2 shows only the significant associations. Fearlessness (T1) negatively predicted warm and positive parenting (T2), whereas it increased the likelihood of punitive parenting (T3), CU traits (T4), and CP (T5). Despite the lag of time, the largest association was between fearlessness and CP. Both T2 warm and positive parenting negatively predicted T3 punitive parenting and T4 CU traits, whereas only positive parenting negatively predicted T5 CP. Warm and positive parenting were positively correlated. Punitive parenting positively predicted T4 CU traits and T5 CP. Finally, CU traits (T4) predicted future CP (T5). 

### 3.3. Indirect Effects

In addition to direct associations, several indirect effects were identified. The total indirect effect from fearlessness to CP through parenting variables and CU traits was significant, *β* = 0.14, *SE* = 0.02, *p* < 0.001. The specific indirect effect that accounted for most of the variance was from fearlessness to CU traits to CP, *β* = 0.11, *SE* = 0.02, *p* < 0.001. The second stronger indirect effect was from fearlessness to punitive parenting to CP, *β* = 0.03, *SE* = 0.01, *p* < 0.01. An additional indirect path from parental warmth to CU traits to CP was identified, *β* = −0.04, *SE* = 0.01, *p* < 0.01, indicating that warm parenting only indirectly influenced CP. Similarly, we found an indirect pathway from positive parenting to CU traits to CP, *β* = −0.03, *SE* = 0.01, *p* < 0.05. In accordance with MacKinnon et al. [72], the identified direct and indirect effects explained 72% of the variance in CP. 

### 3.4. Gender Differences in Structural Associations: Multi-Group Path Model

To test for gender differences, we compared an unconstrained to a constrained model. Findings suggested that the model that freely estimated the associations under study across gender (i.e., unconstrained model) fit the data better than the model that constrained the associations to be equal, Δχ^2^_(21, *N* = 2824)_ = 420.52, *p* < 0.001. Several gender differences were identified, as shown in Figure 2. Fearlessness was more strongly associated with boys’ CP compared to girls. Punitive parenting was only significantly associated with boys’ CP, and warm parenting mainly predicted girls’ CU traits. In terms of indirect effects, we identified two gender-specific pathways: The pathway from punitive parenting to CU traits to CP was only significant for girls (*β* = 0.05, *SE* = 0.01, *p* < 0.001), but not for boys (*β* = 0.01, *SE* = 0.02, *p* = 0.15). The pathway from fearlessness to punitive parenting to CP was only significant for boys (*β* = 0.05, *SE* = 0.01, *p*< 0.001), but not for girls (*β* = 0.01, *SE* = 0.01, *p*= 0.65).

## 4. Discussion

The current study tested the applicability of the InterFear model [6] in a community sample of children in Spain by investigating both direct and indirect effects of fearlessness on CP. Similar to the original study, fearlessness in early childhood was associated with future CP. Furthermore, an indirect pathway through low positive parenting (T2), high negative parenting (T3), and CU traits (T4) was evidenced, supporting the InterFear model. As in the original study, the specific indirect effect from fearlessness to CP via CU traits accounted for most of the variance, pointing to the existence of a temperamental pathway independent of parental variables. Furthermore, we found another pathway that started with low parental warmth instead of fearlessness and influenced engagement in CP through CU traits. Finally, gender differences were identified, suggesting that punitive parenting was only significantly associated with boys CP and that warm parenting mainly predicted girls’ CU traits. Moreover, the pathway from punitive parenting to CU traits to CP was only significant for girls, whereas the pathway from fearlessness to punitive parenting to CP was only significant for boys. Overall, the current study replicates Fanti et al. [6] findings, providing additional support for the InterFear model.

### 4.1. Fearlessness as an Antecedent of CP: The Role of CU Traits

Current findings provide evidence that fearlessness is an early risk factor for the development of CP. It has been proposed that the mechanism connecting fearlessness to future CP is associated with impairments in punishment sensitivity, empathy, and guilt, which put children with a fearless temperament at higher risk for behaviors with negative consequences for themselves or others [8,9,11,14]. In this context, CU traits become relevant, as they encompass low empathy, guilt, and sensitivity to punishment as their defining characteristics [13]. The fact that the indirect effect of fearlessness on CP via CU traits explains most of the variance of the total model provides support for this theoretical proposition. However, CU traits, like any other psychological profile, do not develop in a contextual vacuum. Therefore, the InterFear model takes into account the immediate environment of the developing person (typically their parents) and proposes a person–environment developmental perspective.

### 4.2. InterFear: A Transactional Model

The total indirect effect found in the current study supports the InterFear model, suggesting a potential pathway to CP that includes a dynamic interaction between child and environmental variables: a fearless child reduces parents’ positive parenting behaviors and increases negative parenting, which in turn further contributes to the development of CU traits, setting the stage for the manifestation of CP. Therefore, our findings are in line with transactional models of development (e.g., [74]), which emphasize the importance of understating the complex interplay between a child’s temperament and parenting style. Our findings further support Patterson’s coercion model in terms of the dynamic pathway that leads to CP, which is influenced by both individual characteristics and parental responses [41].

### 4.3. Fearlessness and Parenting

Agreeing with the InterFear framework, fearlessness evokes a parenting style characterized by less warmth and positivity but more punishment. Specifically, the association between fearless temperament and harsh parenting supports the theoretical proposition that parents of under-aroused/fearless children may resort to harsher methods to stimulate enough reactivity in their children so that they conform [31,63,75]. Current findings also provide evidence that the pathway starting with fearlessness leads to low parental warmth, which, as documented in prior work (e.g., [22,23,24]), exacerbates the risk for the development of CU traits and future CP. This finding extends past work, suggesting that low anxiety (akin to fearlessness) is uniquely associated with future CU traits in the context of low parental warmth [34]. Taken together with findings that show that warm parenting from adoptive mothers moderates the genetically influenced effect of fearlessness on CU traits [54], our model highlights the importance of parenting interventions to prevent fearless children from entering a developmental pathway that leads to CU traits and CP.

### 4.4. Additional Pathways to CP: The Role of Parental Warmth

In addition to the indirect pathway from fearlessness to CP, two additional indirect pathways, starting from low parental warmth and positive parenting leading to CP via CU traits, were identified. A similar pathway was also identified in the original study [6] and is in line with genetic and adoption studies suggesting that low parental warmth might be a non-heritable and “causal” risk factor for the development of CU traits [64,65]. Notably, as in the original study, parental warmth was only indirectly influencing CP via CU traits, strengthening the importance of parental warmth for the subgroup of children with high levels of both CP and CU traits [22,24].

### 4.5. Gender Differences

Overall, the InterFear model seems to equally apply to boys and girls. Nevertheless, specific direct and indirect effects of individual and familial variables on future CP were moderated by gender. Specifically, punitive parenting was directly associated with CP only in boys. This is consistent with the original InterFear study [6] and aligns with previous work that has found this direct association to be stronger in boys [76,77]. Low parental warmth directly predicted CU traits only in girls. In addition, only a significant indirect relationship from punitive parenting to CP through CU traits was identified for girls. This finding contradicts prior results suggesting no moderating role of gender in the association between parenting styles, CU traits, and CP [22]. However, it is necessary to conduct further research to clarify this issue since the studies conducted so far are limited. Further, fearlessness was more strongly associated with CP in boys than in girls. This suggests that, although fearlessness is a significant factor in the development of CP in both genders, it may have a greater impact on boys. This is consistent with studies indicating that boys’ increased vulnerability to CP may be due to individual rather than family factors [78]. Finally, a significant indirect path from fearlessness to CP through punitive parenting mainly applied to boys, indicating that parents might be more likely to respond with punishment to boys’ fearlessness.

### 4.6. Strengths and Limitations

The current study’s main strengths are the five time point longitudinal design, the inclusion of both child and familial variables, as well as the use of parent and teacher reports. Nevertheless, the findings of the current study should be interpreted in the context of its limitations. A potential limitation is that parent ratings were mostly based on biological mother reports, which could lead to method and information variance as well as social desirability bias, threatening the validity of the results. Another limitation is that the study was based on a community population. Therefore, in order to draw conclusions about high-risk populations, replication of the current findings in clinical samples (i.e., diagnosis of Conduct Disorder or Oppositional Defiance Disorder) is suggested. Finally, at each time point, we collected data for children with a three-year difference in age. Thus, we did not account for potential developmental differences in these age groups. However, each age group represented a specific developmental period. 

## 5. Conclusions

In conclusion, current findings contribute to an ongoing body of longitudinal research that examines fearlessness as a developmental precursor of CP. Our findings replicate prior work [6], suggesting that a child’s fearless temperament, viewed within a person–environment developmental perspective, increases the likelihood of future CP. Specifically, we provide additional evidence for the developmental pathway proposed by the InterFear model, according to which fearlessness is likely to evoke specific environmental responses (e.g., more negative and less positive parenting), potentially fostering the development of a socio-emotional profile (e.g., CU traits) that exacerbates the manifestation of CP. At the same time, we also provide evidence for additional pathways to CP that are not necessarily related to fearlessness, further informing developmental models of antisocial behavior. For instance, taken together with the findings of our original study, we propose that low parental warmth, which is a well-known risk factor for CP, only indirectly relates to CP via CU traits. 

Our findings can also be important for clinical practice, highlighting the importance of identifying children with fearlessness as a high-risk group and designing specific parenting interventions based on the InterFear model (e.g., increasing parental warmth) to mitigate their risk of developing CP. For example, a recent study found that parenting interventions, which emphasized warmth as well as exchange and sharing of emotions, resulted in increased physiological reactivity to others’ emotions [79]. This finding is of great importance because fearlessness is related to lower physiological reactivity [13], suggesting that in addition to strengthening the parent–child relationship, parenting interventions can also diminish the physiological deficits associated with fearlessness. Additionally, our findings indicate that fearlessness starts influencing the child’s development before the age of 6, and interventions implemented early in development might have a higher likelihood of success.

## Figures and Tables

**Table 1:** Descriptive Statistics and Correlations among the Main Study Outcomes.

	Fearlessness (Parent-T1)	Fearlessness (Teacher-T1)	Warm Parenting (T2)	Positive Parenting (T2)	Punitive Parenting (T3)	CU Traits (T4)	CP (Parent) (T5)	CP (Teacher) (T5)
Fearlessness (teacher-T1)	0.23 **							
Warm parenting (T2)	−0.04	−0.05 *						
Positive parenting (T2)	−0.04	−0.05 *	0.62 **					
Punitive parenting (T3)	0.06 *	0.13 **	−0.06 *	0.02				
CU traits (T4)	0.12 **	0.14 **	−0.17 **	−0.17 **	0.14 **			
CP (parent) (T5)	0.22 **	0.19 **	−0.16 **	−0.19 **	0.19 **	0.45 **		
CP (teacher) (T5)	0.32 **	0.20 **	−0.02	−0.01	0.13 **	0.19 **	0.33 **	
*Descriptive:*								
Mean	1.78	1.30	4.67	4.46	2.08	1.24	1.44	1.32
SD	0.66	0.55	0.38	0.34	0.35	0.37	0.45	0.50

Note. T = Time; ** = *p* < 0.001; * = *p* < 0.05.

## Data Availability

The raw data supporting the conclusions of this article will be made available by the authors on request.
